# Notice of Correspondence

**Published:** 1862-04

**Authors:** 


### NOTICE OF CORRESPONDENCE.

Our correspondent, "O. U. C." *emphatically* denies in this number, that he had any thought or intention of claiming "superior ability" for his favorite faculty. Well, then, what is between us? for we never intended to, and never did, claim superior ability for anybody else. We disclaimed *inferior* ability for the other faculties. That is all. And no torture, by paraphrase or otherwise, can make our language say any thing else. Our correspondent's paraphrases are ungenerous and unjust; and as wide of the mark as his reference to the man that trusted to ravens. Still he is not altogether uncandid; for he admits that his manner of speaking may have induced the inference that he claimed *superior* ability for the faculty there. Exactly! That is just the point. To a reader who could not identify our correspondent through his *nom de plume*, the case appeared thus:—

Here is a communication from a special correspondent of this journal whose present publisher and chief editor is a professor in a dental college. This correspondent resides in the vicinity of a rival school. From the tenor of his remarks, it is to be inferred that he is a member of the faculty, a *special agent*, or a TRUSTEE of that institution. He appears to be intelligent and *sharp*, and, therefore, concludes he can make his position as correspondent profitable to *his* school. So he writes a beautifully worded *puff* of his favorite, and devotes the remainder of his space to decrying a member of a *rival faculty*. He is shrewd enough to know that *ten* will read the puff in his correspondence, where *one* will read a regular notice of his school in the advertising department. This plan, too, had the merit of economy; for the puff, if admitted thus, would *cost nothing*, and might, possibly, do away with the necessity of legitimate advertising in the same journal, which, of course, would be attended with trifling expense.

This is the view of the whole matter actually taken by a disinterested party. Indeed, no other could be taken by any one not admitted behind the scenes. But this is all set aside now, by the *emphatic* disclaimer of our correspondent.

Our correspondent concludes these "solemn exercises" by calling this a "gratuitous" discussion, from which we infer that he is not paid for his services as a puffer; but we insist he should be. He also calls it "unpleasant," to which we can only say, in the language of the heroic Sigel, "If you not like the war, *what you make him for?*"

But as *our* correspondent, to all appearance, has undertaken to act as *agent* and champion of the Pennsylvania College, we are ready to propose a compromise. If he will allow the other schools to hold, with reference to his favorite, the sentiments of Job xiii. 2, we will subscribe to Job xii. 2, have our signature duly attested, the document appropriately framed, and forwarded to him to be hung up in front of the rostrum, in the lecture hall of his favorite school. W.

